# Evaluation of the effectiveness of the photobiomodulation in the treatment of dentin hypersensitivity after basic therapy. A randomized clinical trial

**DOI:** 10.4317/jced.53635

**Published:** 2017-05-01

**Authors:** Cristina García-Delaney, Daniel Abad-Sánchez, Josep Arnabat-Domínguez, Eduard Valmaseda-Castellón, Cosme Gay-Escoda

**Affiliations:** 1 DDS. Master of Oral Surgery and Orofacial Implantology, School of Dentistry of the University of Barcelona, Spain; 2 DDS. Professor Master Degree program in Oral Surgery and Implantology, School of Dentistry of the University of Barcelona. Researcher of the IDIBELL Institute, Barcelona, Spain; 3 MD, DDS, PhD, Associate Professor of Oral Surgery. Master’s Degree program in Oral Surgery and Implantology, School of Dentistry, University of Barcelona. Researcher of the IDIBELL Institute, Barcelona, Spain; 4 DDS, PhD, Professor of Oral Surgery. Master’s Degree program in Oral Surgery and Implantology, School of Dentistry, University of Barcelona. Researcher of the IDIBELL Institute, Barcelona, Spain; 5 MD,DDS, MS, PhD, EBOS, OMFS. Chairman and Professor of Oral and Maxillofacial Surgery, School of Dentistry, Barcelona. Director of the Master’s Degree Program in Oral Surgery and Implantology (EHFRE International University/FUCSO). Coordinator/Researcher of the IDIBELL Institute. Head of the Oral Surgery, Implantology and Maxillofacial Surgery Department of the Teknon Medical Center, Barcelona, Spain

## Abstract

**Background:**

Dentin hypersensitivity (DH) in one of the most common causes of patient discomfort in the general population and its prevalence is higher in patients who have received basic or surgical periodontal treatment. Efficiency of the diode laser with different wavelengths has been studied by several authors, showing an improvement rate of the DH between 60-98%. The aim of the present study was to evaluate the effect of photobiomodulation (PBM) treatment on the reduction of DH after non surgical periodontal treatment.

**Material and Methods:**

A randomized split mouth clinical trial was performed involving 30 patients (120 teeth) diagnosed with DH after scaling and root planning. Two teeth of the experimental side were treated with the laser and 2 teeth of the control side were treated without activating the laser. The laser treatment parameters for each tooth were 660nm, 200mW, CW, illuminated area 1.15cm2, 173mW/cm2, 60 seconds, 12 J, 10.4J/cm2. Age, gender, smoking, plaque index, gingival recession, probing and VAS (for tactile and thermal stimulation) were registered before the laser treatment, immediate post treatment (after 2 minutes), 2 weeks, 1 month and 2 months after treatment.

**Results:**

There was significant difference (*p*<0.01) in discomfort to thermal and mechanical stimulation between the control and diode laser treatment sites at all evaluation periods. The level of discomfort decreased immediately following diode laser therapy, and continued to demonstrate a decrease for the duration of the study. All teeth remained vital after laser treatment, without adverse reactions or complications.

**Conclusions:**

The PBM can be used to reduce DH without detrimental pulpal effects.

** Key words:**Dental hypersensitivity, laser, diode laser, photobiomodulation.

## Introduction

Dentin hypersensitivity (DH) is defined as a short, intense pain that originates in the exposed dentin in response to a chemical, thermal, evaporative, tactile or osmotic stimulus and cannot be attributed to other dental defect or pathology (1).

Over 90% of tooth surfaces with DH are located at the gingival margin. The origin of the injury may be due to loss of enamel or gingival recession exposing the root surface.

It may be present in a region of the mouth, in several teeth or affect a single tooth. The teeth most frequently involved are the canines and premolars (1-4).

DH is one of the most common causes of patient discomfort in the general population. Its prevalence varies considerably, ranging between 4 and 57%, being more frequent in patients aged between 30 and 40 years (1).

Its prevalence is higher in patients with periodontal disease (60-98%) or in patients who have received basic periodontal treatment (scaling and root planning) or surgical treatment. According to a systematic review of Von Troil *et al.* (5), the prevalence of DH after periodontal treatment was 54-55%. DH intensity peaked between the 1st and the 8th week post-treatment.

The instrumentation of root surfaces in periodontal therapy exposes dentinal tubules to the oral environment, making the dentin susceptible to bacterial, chemical and mechanical stimuli. This exposure increases the hydraulic conductance within the exposed tubules causing a painful sensation. Although periodontal therapy is an important etiologic factor in the cause of DH, scaling and root planning exposed root surfaces triggers no hypersensitivity in some patients (6).

Electron microscopy studies show that the areas of dentin hypersensitivity have multiple open dentinal tubules. In contrast, non-sensitive dentin areas on the surface show that most tubules are sealed (7). These findings may explain why not all patients with exposed root surfaces have DH.

Until now various substances have been tested for the treatment of DH with varying degrees of success. There are several treatment options, applied by the patient or by the practitioner (application of topical agents potassium nitrate, oxalate, fluoride, adhesives or resins etc.), or other complex methods as iontophoresis or the laser (8).

The first laser used for the treatment of DH was described by Matsumoto *et al.* (9) in 1985 with the use of Nd: YAG. Since then, several studies have been published, with a significant increase in the last 10 years, showing the growing interest in this topic.

Various laser types have been tested for DH treatment, including Nd:YAG and Er:YAG, CO2, He-Ne, and diode (ie, GaAlAs) lasers, with various energy settings and with wavelengths ranging from 632.8 nm (He-Ne) to 10,600 nm (Er:YAG, CO2)(10).

For low output-power lasers (diode laser=780-900 nm or He-Ne lasers=632.8 nm) the desensitizing effect seems to be related to laser activity at the nervous level. It has been shown that can increase the metabolic activity odontoblasts and mediate an analgesic effect related to depressed nerve transmission by inhibiting fast axonal flow and reducing amplitude in superficial C fibers and Aδ fibers (11). Nevertheless, the desensitizing effect of the middle output-power lasers (Nd:YAG, CO2, and Er:YAG) could be related to an interaction with the dental pulp, that causes a photobiomodulating effect, increasing the cellular metabolic activity of the odontoblasts and occluding the dentinal tubules with the intensification of tertiary dentine production (12). Arany *et al.* (13) evaluate the ability of low power laser therapy to direct differentiation of dental stem cells for dentin regeneration, and investigate the precise molecular mechanisms involved in the process. Adult human dental stem cells in the tooth pulp express characteristic stem cell surface markers and are capable of multiple lines differentiation, making them key players in tooth regeneration.

Ladalardo *et al.* (2) noted that the degree of pain reduction was greater in patients with an age range between 25 and 35 who belong to the group of 36 to 45 years, a fact attributed to the morphological changes that occur in dentine structure over the years.

Lasers are commonly used in the treatment of dentine hypersensitivity, and their effectiveness ranges from 5-2% to 100%, depending on the laser type and parameters used. Three wavelengths (780, 830 and 900 nm), all within the infrared spectrum of GaAlAs diode laser, have been used for the treatment of DH but the use of red wavelength diode laser has also been reported (8). Efficiency of the diode laser with different wavelengths has been studied by several authors, showing an improvement rate of the DH between 60-98% (2).

For this reason, our objective in this study was, to assess the effectiveness of the diode laser 660nm, 200mW, CW, illuminated area 1.15cm2, 173mW/cm2, 60 seconds, 12J, 10.4J/cm2 for the treatment of exposed surfaces with DH after non surgical periodontal treatment.

## Material and Methods

-Patient selection

A split mouth randomized clinical trial was performed involving 30 patients (120 teeth, 60 control and 60 experimental). 10 patients were treated in the Periodontal Pathology and Surgery Unit belonging to the Oral Surgery and Implantology Master Degree program of the University of Barcelona and 20 patients were treated in a a private clinic of the same area. Patients were treated with scaling and root planing and subsequently referred for PBM if diagnosed with dentin hypersensitivity in at least 2 teeth at different quadrants. Exclusion criteria were.

1. Any desensitizing treatment (current or last month).

2. Pregnancy.

3. Eating disorders (bulimia, etc.) or diet that cause erosion and / or tooth wear.

4. Orthodontic treatment.

5. Teeth whitening in the past 3 months.

6. Teeth with large fillings or reconstructions affecting the assessment area.

7. Teeth with fractures, cracks or untreated caries.

8. Non-vital teeth or pulpal pathology.

9. Parafunction.

All patients were informed of the nature and objectives of the study, and signed consent prior to inclusion in the study. The institutional review board (Ethical Committee of Clinical Investigation, University of Barcelona Dental School) reviewed and approved the study protocol.

Following the baseline examination, each side was randomly allocated either to the treatment or the control side with a series of random numbers.

**Figure 1:**
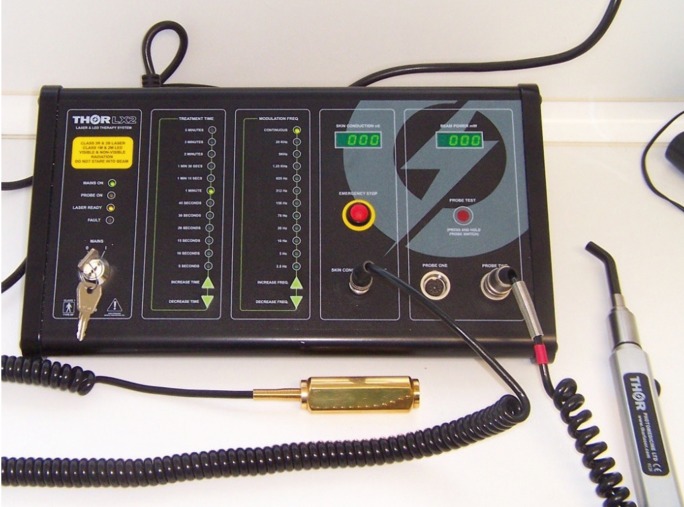
Diode laser THOR LX2 ® (Thor Photomedicine Ltd, Chesam, London).

Each patient received laser treatment on 2 teeth of the experimental side with the laser THOR LX2 (THOR Photomedicine Ltd, Chesham, UK) (Fig. 1) at a 5 mm distance, with oscillating movements, wavelength 660nm, power 200mW, continuous mode, illuminated treatment area 1.15cm2, irradiance 173mW/cm2, irradiation time 60 seconds, energy 12 Joules, fluence 10.4J/cm2. In the control side treatment of 2 teeth was simulated without activating the laser. To maintain 5 mm distance, the operator essay the position previously, however this position can be difficult to reproduce in all cases.

-Clinical parameters

Age, gender, smoking and plaque index were gathered. For the 2 test teeth and the 2 control teeth: recession (the highest point), probing (of the highest point of recession) and the degree of dentin hypersensitivity using visual analog scales (VAS) for tactile stimulation (touching the tooth neck with a sharp dental probe) and thermal stimulation (with an air jet from the syringe dental chair, isolating adjacent teeth with cotton rolls).

The VAS from 0 to 100 (0 = no pain, 100 = maximum tolerable pain) were recorded before the laser treatment, immediate post treatment (after 2 minutes), 2 weeks, 1 month and 2 months after treatment for both stimuli.

-Data analysis

Statistical analysis was carried out with SPSS15.0 using repeated measures ANOVA. The main outcome variables were the VAS for thermal and tactile stimuli.

## Results

Patient age ranged from 19 to 67, with a mean age of 41.8 years (9 males and 21 females). The main of cigarettes/day was 3.26 (range 0-20 cigarettes), 22 patients were smokers (73.3%) and 8 non smokers (26.7%). The plaque index (O´Leary index) was 3.18 (range 0-77). The results of the laser application can be seen in  Table 1. And a more detailed tables show a distribution of estimated marginal means of VAS for thermal ( Table 2) and tactile stimulation ( Table 3) for each patient.

**Table 1:**
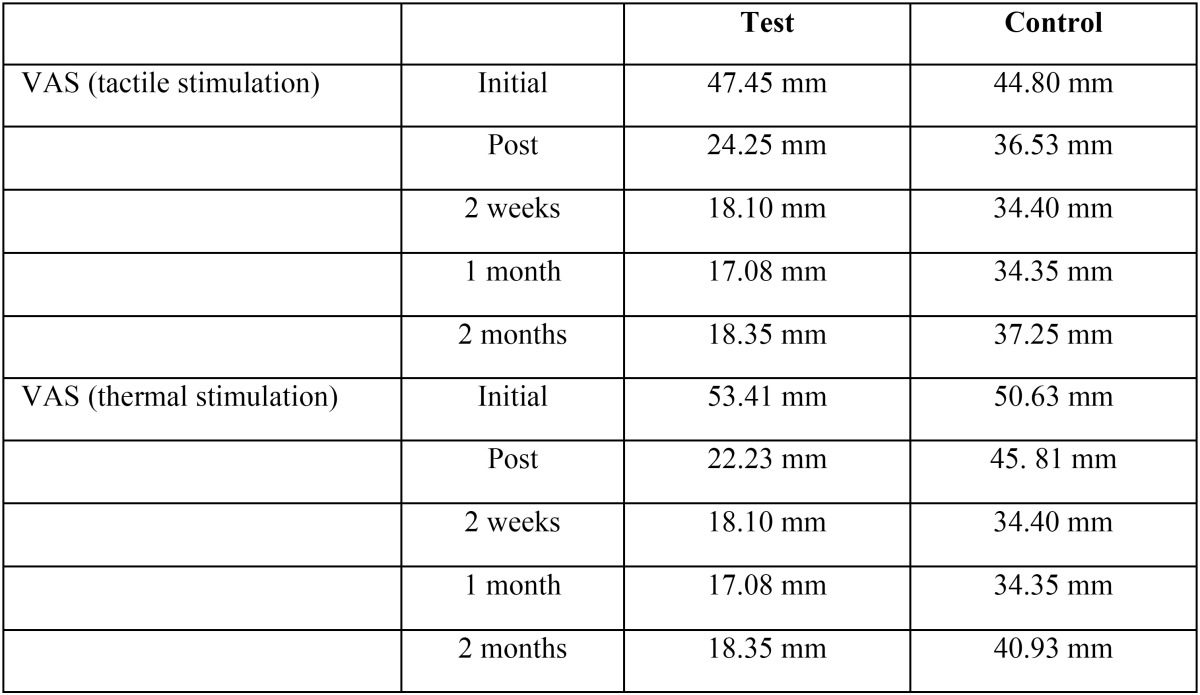
Distribution of estimated marginal means of VAS for tactile and thermal stimulation in both groups.

**Table 2:**
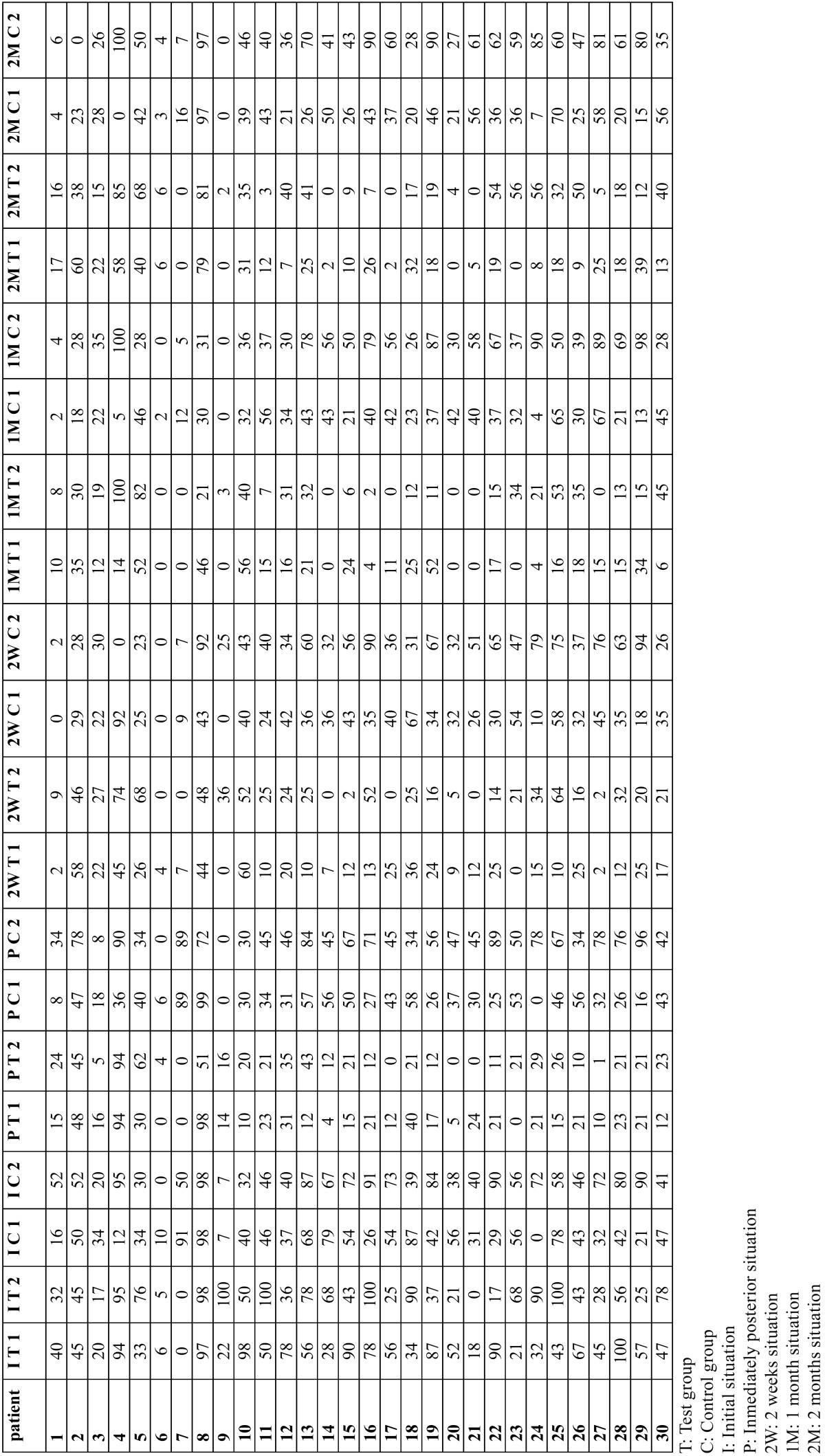
Distribution of VAS for thermal stimulation for each patient and tooth.

**Table 3:**
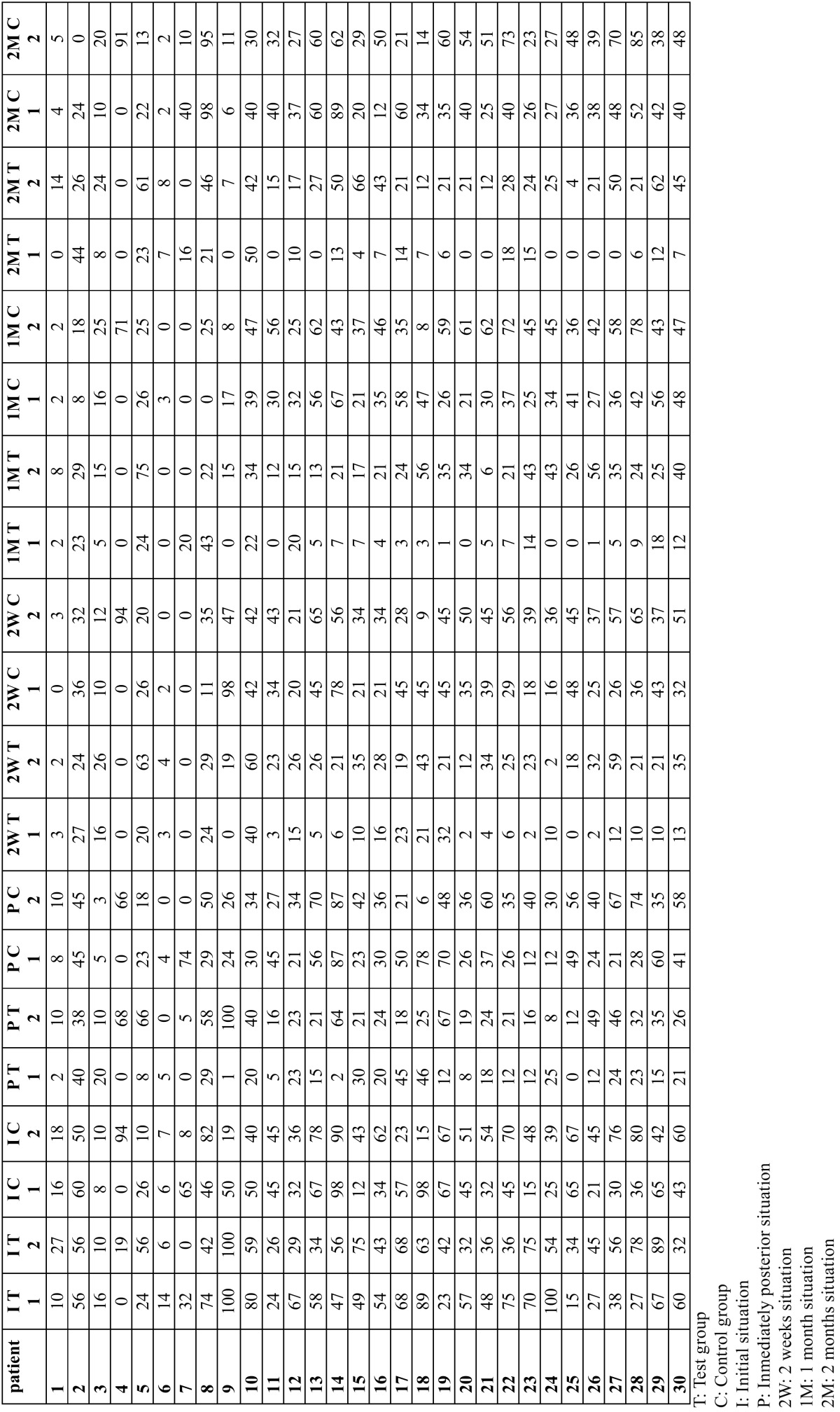
Distribution of VAS for thermal stimulation for each patient and tooth.

**Figure 2:**
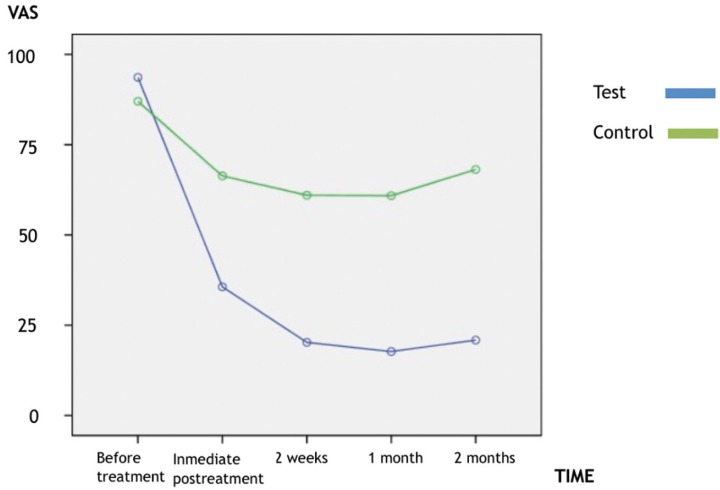
Estimated marginal means of VAS to tactile stimuli.

**Figure 3:**
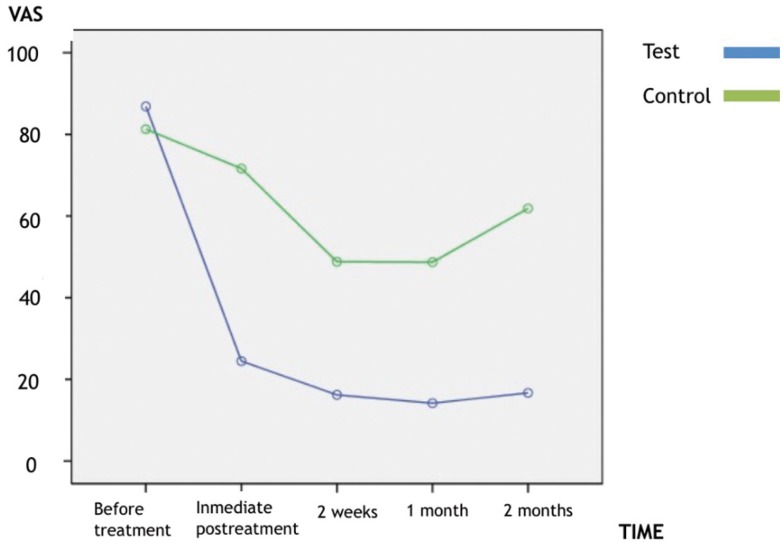
Estimated marginal means of VAS to thermal stimuli.

The level of discomfort elicited by thermal and mechanical stimulation decreased significantly (*p* < 0.01) immediately following diode laser therapy, and continued to demonstrate a decrease for the duration of the study. There was significant difference (*p* < 0.01) in discomfort to thermal and mechanical stimulation between the control and diode laser treatment sites at all evaluation periods (Figs. 2,3).

All teeth remained vital as measured by the 2 methods of stimulation. There were no adverse effects of diode laser treatment and no complications.

## Discussion

The management of DH can be performed at home with desensitizing toothpastes, mouthwashes and chewing gums containing potassium salts (potassium nitrate, potassium chloride or potassium citrate) that are thought to diffuse along dentinal tubules and decrease the excitability of dental nerves by altering their membrane potential. In office treatments, like topically applied desensitizing agents (fluoride, potassium nitrate, oxalate, calcium phosphates), adhesives and resins and other procedures like iontophoresis and lasers (1).

The action mechanisms of diode lasers in dentin hypersensitivity treatments have been suggested by several authors. This type of low output power lasers mediate an analgesic effect related to depressed nerve transmission, but this analgesic effect usually only last 24 hours, there are also regenerative effects (odontoblasts). According to experiments using the diode laser at 830 nm, this effect is caused by blocking the depolarization of C-fibers afferents. Diode laser irradiation at a maximum power of 60 mW does not affect the enamel or dentin surface morphologically, but a small fraction of the laser energy is transmitted through enamel or dentin to reach the pulp tissue (14,8).

Dahnhardt *et al.* (15) mentioned that the two main treatment options for dentin hypersensitivity are desensitization of the nerve and the mechanical occlusion or covering of the dentin tubules. However, the study period was not long enough to examine such an effect in this study. According to Corona *et al.* (16), the low-level GaAlAs laser showed improved results for treating teeth with a higher degree of sensitivity. However, Kimura *et al.* (8) concluded that in general, the efficiency for the treatment of dentin hypersensitivity using lasers is higher than in other methods, but in severe cases, it is less effective. It is necessary to consider the severity of dentin hypersensitivity before laser use (8,17). The patient groups used in our study had moderate dentin sensitivity.

PBM is the application of light (usually delivered via a low power laser of light-emitting diode; LED) to promote tissue repair, reduce inflammation or induce analgesia. PBM differs from photodynamic therapy (PDT), which utilizes light indirectly to trigger photosensitive dyes to produce bactericidal molecules that kill infecting microbes that cause disease. In contrast, PBM uses the action of light and light alone to directly stimulate host cells in order to reduce inflammation, relieve pain and/or promote wound healing. For PBM to be effective, the applied irradiation parameters including wavelength, power, irradiance, exposure time, and pulse need to be applied within limits (18).

The laser or LED devices applied in PBM typically emit in the 600-1000 nm spectrum range (red to near infrared). Other wave-lengths outside the 650-850 nm spectrum can have similar effects they do not penetrate the tissues as well as those in the red and near-infrared range (19).

In the present study, patients who complained of DH in the follow up after basic periodontal therapy were evaluated for tactile and thermal nociceptive sensitivity.

The effectiveness of DH treatment with PBM, with different wavelengths, has been reported in various clinical studies. Gerschman *et al.* (20) found that the sensitivity to thermal stimuli was reduced by 67% and to tactile stimuli by 65%. In our study was reduced 32% and 36% respectively. Should consider that dosimetry and the study design are different.

Ladalardo *et al.* (2) concluded that the 660 nm red laser (35 mW) was more effective than the 830 nm infrared laser. Yamaguchi *et al.* (21) used the GaAlAs diode laser with 790 nm (30 mW) and reported effective improvement of 60% in the laser group.

Others evaluated the effectiveness of the clinical use of diode lasers for the treatment of dentin hypersensitivity and reported their use as effective in reducing initial hypersensitivity. In our study also found the treatment with the diode laser effective in a short-term time period, but we found significant differences in all evaluation periods too. A trial (22) demonstrated that GaAlAs laser had a significantly greater immediate response in treating DH. The effect had become obvious at 15 min, and it remained stable until 2 months. On the contrary, Vieira *et al.* (23) compare the immediate and 3-month desensitizing effects of a GaAlAs laser (660 nm), a 3% potassium oxalate gel and a placebo gel. The VAS scores for air blast and tactile stimuli manifest significant reductions in DH, but there was no significant difference among the two groups.

The mean age was 41.8 years for this study. Dentin hypersensitivity is prevalent among a large portion of individuals 30-40 years of age. The results obtained in the study of Ladalardo *et al.* (2) made evident that satisfactory desensitizing levels were only found in patients ranging in age between 25 and 35 years and a higher degree of desensitization was also observed at 15 and 30 minutes after irradiation. In our results, the age in not an important factor, and the higher degree of desensitization was observed at the first month. Should considerer that both studies are different about the method, equipment and power density. We use more power and less exposure time getting significant results too.

Our results indicated significantly decreased pain scores at post-treatment evaluations for red wavelength low-intensity diode laser. No relapse of sensitivity during the post-treatment evaluation period was detected for this treatment. The untreated control side showed higher degrees of discomfort in follow-up sessions, although it followed a similar pattern of reduction of hypersensitivity than the test side.

Sicilia *et al.* (22) concluded that the application of diode laser has shown efficacy in rapid dentin hypersensitivity reduction compared to placebo laser in periodontal patients. And mentioned that the application of a diode laser at a wavelength of <780 nm and at an output power below 30 mW, with an application time of <3 min, is a safe treatment with regard to pulp.

Tengrungsun *et al.* (24) concluded that the reduction of dentin hypersensitivity by dentin bonding agent was significantly superior to GaAlAs laser (*p* < 0.05) and no significantly additional reduction in level of hypersensitivity from day 15 to 30 was observed. On the other hand, Pesevska *et al.* (25) compare the effectiveness of low-level laser irradiation to traditional topical fluoride treatment for DH following scaling and root planning. The experimental group treated with diode laser show a complete absence of pain in 86.6% of patients and only in 26.6% in the fluoride treated group. Another study with a longer follow-up (6 months) (26) compare low-power laser at different dosages (30 mW, 10 J/cm2, 9s, 810 nm versus 100 mW, 90 J/cm2, 11s, 810 nm), a desensitizing agent, and associations. All treatments performed were efficient in the reduction of cervical dentinal hypersensitivity and this effect was maintained stable until 6 months. But the treatments performed with low-power laser at a low dose were shown to be more efficient in diminishing pain more quickly when compared with a high dose. Nevertheless, both were equally effective in the long term.

Another study using 660 nm laser irradiation (27) supports our conclusions taking into account differences in dosimetry (25 mW, 11.36 mW/cm2, 3min.), follow-up (7 days) and treatment (DH following periodontal flap surgery). These authors showed statistically significant differences in VAS for pain on the test site compared on control site and on day 7 compared with day 1 of treatment.

The data in this randomized controlled trial suggest that the use of red wavelength diode laser may be effective in the short-term treatment of cervical dentinal hypersensitivity.

Long-term evaluation of effectiveness of these treatments needs to be carried out to know the stability of the results and if relapse occurs.
